# Epidemiological and Molecular Characterization of a Mexican Population Isolate with High Prevalence of Limb-Girdle Muscular Dystrophy Type 2A Due to a Novel *Calpain-3* Mutation

**DOI:** 10.1371/journal.pone.0170280

**Published:** 2017-01-19

**Authors:** Carlos A. Pantoja-Melendez, Antonio Miranda-Duarte, Bladimir Roque-Ramirez, Juan C. Zenteno

**Affiliations:** 1 Department of Genetics-Research Unit, Institute of Ophthalmology “Conde de Valenciana”, Mexico City, Mexico; 2 Department of Genetics, Instituto Nacional de Rehabilitacion, Mexico City, Mexico; 3 Pharmacobiology Department, CINVESTAV-Sede Sur, Mexico City, Mexico; 4 Department of Biochemistry, Faculty of Medicine, UNAM, Mexico City, Mexico; University of Minnesota, UNITED STATES

## Abstract

Limb-Girdle Muscular Dystrophy type 2 (LGMD2) is a group of autosomally recessive inherited disorders defined by weakness and wasting of the shoulder and pelvic girdle muscles. In the past, several population isolates with high incidence of LGMD2 arising from founder mutation effects have been identified. The aim of this work is to describe the results of clinical, epidemiologic, and molecular studies performed in a Mexican village segregating numerous cases of LGMD2. A population census was conducted in the village to identify all LGMD affected patients. Molecular analysis included genome wide homozygosity mapping using a 250K SNP Affymetrix microarray followed by PCR amplification and direct nucleotide sequencing of the candidate gene. In addition, DNA from 401 randomly selected unaffected villagers was analyzed to establish the carrier frequency of the LGMD2 causal mutation. A total of 32 LGMD2 patients were identified in the village, rendering a disease prevalence of 4.3 (CI: 2.9–5.9) cases per 1,000 habitants (1 in 232). Genome wide homozygosity mapping revealed that affected individuals shared a 6.6 Mb region of homozygosity at chromosome 15q15. The identified homozygous interval contained *CAPN3*, the gene responsible for LGMD2 type A (LGMD2A). Direct sequencing of this gene revealed homozygosity for a novel c.348C>A mutation (p.Ala116Asp) in DNA from all 20 affected subjects available for genetic screening, except one which was heterozygous for the mutation. In such patient, a heterozygous c.2362AG>TCATCT deletion/insertion was recognized as the second *CAPN3* mutation. Western blot and autocatalytic activity analyses in protein lysates from skeletal muscle biopsy obtained from a p.Ala116Asp homozygous patient suggested that this particular mutation increased the autocatalytic activity of CAPN3. Thirty eigth heterozygotes of the p.Ala116Asp mutation were identified among 401 genotyped unaffected villagers, yielding a population carrier frequency of 1 in 11. This study demonstrates that a cluster of patients with LGMD2A in a small Mexican village arises from a novel *CAPN3* founder mutation. Evidence of allelic heterogeneity is demonstrated by the recognition of an additional *CAPN3* mutation in a single affected. Our study provides an additional example of genetic isolation causing a high prevalence of LGMD and of successful molecular characterization of the disease by means of homozygosity mapping. The identification of a very high carrier frequency of the LGMD2-causing mutation has implications for more rational genetic counseling in this community.

## Introduction

The muscular dystrophies encompass a clinically heterogeneous group of inherited disorders characterized by progressive weakness and degeneration of the skeletal muscles. The distribution and severity of muscular affectation in these conditions as well as their age of onset is highly variable. In some specific forms of the disease, non-skeletal muscles such as cardiac and respiratory muscles could also be affected and other organs such as brain and eyes can be involved. All forms of muscular dystrophy aggravates as muscles progressively degenerate and weaken, and most affected individuals eventually lose their ability to walk [1]. Muscular dystrophies are caused by mutations in any of the dozens of genes encoding proteins needed for muscle integrity and function and are among the most genetically heterogeneous human conditions with up to 165 muscular dystrophies and myopathies-causative genes listed in a recent review [2].

The disease can be subdivided into several groups in accordance with the distribution of predominant muscle weakness and age of onset. Limb-girdle muscular dystrophies (LGMD) are a group of disorders that primarily affects the proximal muscles of the hip and shoulder girdles. Initially described as a distinct clinical phenotype, they are now recognized as a highly heterogeneous group of myopathies that vary in severity and may affect individuals of all ages from childhood through adulthood [3–6]. Depending on their inheritance pattern LGMD are classified into LGMD1, of autosomal dominant transmission, and LGMD2, of autosomal recessive inheritance. A letter is added to each type of LGMD to indicate the order of discovery of each individual gene (e.g. LGMD1A, LGMD1B, LGMD1C, etc.) [4,6,7]. Of 31 LGMD subtypes recognized to date, there are 8 LGMD1 and 23 LGMD2 types [5,6]. The LGMD2 forms are much more common with a cumulative prevalence of 1:15,000 [8], but with differences among ethnic groups depending on the carrier distribution and the degree of consanguinity. LGMD2A seems to be the most frequent type of LGMD worldwide [9–11]. As the relative frequency of the different forms of LGMD varies considerably among populations [12–15], it is recommended to consider the ethnic origin of patients for aiding in the differential diagnosis of disease subtypes. Regrettably, the wide variation in phenotypic expression of the LGMD2 along with its remarkable locus heterogeneity, greatly complicates the clinical and molecular diagnoses in patients suffering from the condition [16–18].

In this work, the genetic and epidemiological characterization of an isolated Mexican population segregating numerous cases of LGMD2 is described. Genome wide homozygosity mapping was used to identify the involved locus and a molecular epidemiologic study was conducted to screen a large unselected sample of the residents to establish the carrier frequency of the responsible mutation. Our study provides the first epidemiologic and molecular characterization of LGMD2A in Mexico and offers another example of genetic isolation producing a high prevalence of autosomal recessive LGMD.

## Materials and Methods

### Recruitment of patients

The study was conducted in a small village located in the state of Tlaxcala in central Mexico. A census of the whole population was accomplished by one of the authors (C. P-M) visiting all households, interviewing household members for recording information about muscular diseases, abnormal gait, or muscle weakness, as well as for collecting biological samples. The investigation was approved by the Research Committee of the Institute of Ophthalmology “Conde de Valenciana” and adhered to the tenets of Helsinki’s declaration. All patients gave their written consent before samples collection.

### Clinical examination of affected individuals

The patients were clinically examined for diagnosis of LGMD. The individuals described in this manuscript have given written informed consent (as outlined in PLOS consent form) to publish these case details. According to the diagnostic criteria proposed by the European Neuromuscular Centre Workshop [19], cases were classified as probable LGMD if they had adolescent (after 10 years of age)- or adult-onset muscle weakness with progressive weakness affecting primarily or predominantly the pelvic and/or shoulder girdle muscles, absence of vertical pattern of inheritance, presence of winging scapulae, no facial, oculo-motor, cardiac or predominantly distal involvement and absence of early contractures. Patients with onset before the age of 8 years or with intellectual discapacity were excluded. Muscle biopsies were performed in some patients several years ago but their results were unavailable at present. Detailed family history was recorded for recognition of affected relatives living outside the village and which could not be examined.

### Genomic DNA isolation

Genomic DNA was isolated from buccal cells using the Gentra Puregene buccal cell kit (Qiagen, Valencia, CA) following the manufacturer’s recommendations.

### Genome wide SNP homozygosity mapping

A genome-wide linkage scan using an Affymetrix 250K *Nsp1* single nucleotide polymorphism (SNP) mapping array (Affymetrix, Inc., Santa Clara, CA) was undertaken in samples from three muscular dystrophy patients to identify shared regions of homozygosity, as previously described [20]. Briefly, 240 ng of pooled DNA (80 ng from each patient) were first digested with the *Nsp1* restriction enzyme (New England Biolabs, Boston, MA) and then ligated to adaptors. Each *Nsp1* adaptor-ligated DNA was amplified in three 100 μl PCR reactions using AmpliTaq Platinum (Clontech Laboratories, Inc., Palo Alto, CA). Fragmented PCR products were then labeled, denatured and hybridized to the array following washing and staining steps on the Affymetrix GeneChip Fluidics Station 450. Fluorescence intensities were quantified with an Affymetrix array scanner 3000–7G and the data were collected by the Affymetrix GeneChip Operating Software (GCOS) 1.4 version. Genotypes were generated using the GTYPE software for BRLMM analysis using default settings. Then, the HomozygosityMapper software was used to analyze the genotypes and for the identification of regions of homozygosity >2 Mb [21]. Candidate disease genes located within the homozygous intervals were identified using GeneDistiller software [22].

### PCR amplification and mutational analysis

Mutations in Calpain 3 (*CAPN3*) were screened by direct sequencing using primer pairs for the 24 coding *CAPN3* exons. All exons were amplified by PCR using Hotstart Taq polymerase (Qiagen). Oligonucleotides sequences and amplification conditions are available on request. PCR products were digested with ExoSAP-IT (GE Healthcare, Chalfont St Giles, UK) and sequenced using the BigDye Terminator Cycle Sequencing kit (Applied Biosystems). Amplicons were analyzed by capillary electrophoresis on an automated sequencer 3130 (Applied Biosystems) and the obtained DNA sequences were manually compared with wild type gene sequences.

### *In silico* analysis

Polyphen-2 (Polymorphism Phenotyping v2) and SIFT (Sorting Intolerant From Tolerant) algorithms were employed to predict pathogenicity of the identified mutation. Phylogenetic conservation of the protein residue affected by the mutation was evaluated by means of Polyphen-2.

### CAPN3 western blots and autocatalytic assay

Slices of skeletal muscle biopsy of one LGMD2A patient and frozen skeletal muscle as normal control were dissolved in 100μl of saline solution and incubated at room temperature for 20 min before addition of EDTA 0.1M. Prior to sonication, the samples were resuspended in Laemmli 2X buffer (Sigma-Aldrich, Milwaukee, WI, USA), heated for 3 min at 95oC, and centrifuged. Protein extracts were run on 10% SDS-PAGE for 3hrs at 100V and electroblotted to PVDF membrane (Millipore, Milford, MA, USA). Blots were blocked with 5% low-fat milk powder and then incubated with monoclonal antibodies against Calpain-3 (Calp3c/12A2, diluted 1:50; Vector Laboratories, Burlingame, CA, USA) and glyceraldehyde-3-phosphate dehydrogenase (GAPDH) (ab2302, diluted 1:300; Millipore) as internal control. Immunoreactive bands were visualized using a peroxidase-conjugated goat anti-mouse secondary antibody (Diluted 1:5000; Jackson Immunoresearch Laboratories, West Grove, PA, USA).

### Estimation of *CAPN3* mutation carrier frequency

The carrier frequency of the LGMD causal mutation was established by analyzing DNA from 401 randomly selected healthy inhabitants of the village. Sample size was calculated following the formula for an infinite population (95% confidence, 80% of power). As the carrier frequency was unknown, a 0.5 value was used for this calculation. Genotyping was performed using direct nucleotide sequencing, as described above.

## Results

### Demographic and clinical data

**Fig 1:**
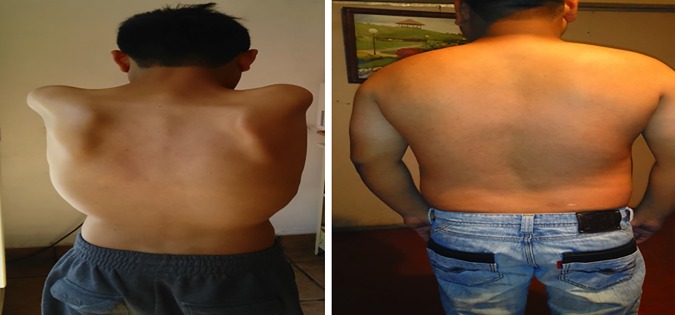
Variable clinical phenotype in LGMD2 patients. (A) Severe winged scapulae are evident in a 22-year- old male patient. (B) Dorso-lumbar scoliosis is observed in a patient aged 25 years.

The village population census indicated that the community was populated by 7,438 individuals from the mestizo population and the Nahuatl indigenous group.Males represent the largest proportion of the population (51.71%) and the age group with more members was 25–44 years (29.3%) (see S1 Fig for data on population structure). From individual household interviews, a total of 32 patients fulfilling clinical criteria for LGMD were identified. Of them, 20 were diagnosed after clinical examination by us while the remaining 12 were first degree relatives affected by the same muscle disease but that were living outside the village and for this reason were diagnosed as LGMD patients merely by family report. Thus, considering only the 20 cases living in the village, a disease prevalence of 2.6 (CI: 1.6–4.0) LGMD2 cases per 1,000 habitants (or 1 in 384) was established. The 32 affected individuals were distributed in 16 family trees (8 families contained multiple affected individuals while the remaining 8 exhibited a single affected individual) following a clear autosomal recessive pattern of transmission. On average, LGMD patients (n = 32) started symptoms at 17.4 ± 3.15 years. The earliest age of symptoms onset was 14 years while the latest one was 30 years. Clinical picture of patients was characterized by weakness and wasting of the muscles of the pelvic girdle with subsequent involvement of the shoulder muscles. Physical anomalies as winged scapulae (Fig 1A), mild scoliosis (Fig 1B), pseudohypertrophy of gastrocnemius, and abnormal gait were also present (Table 1). Support for walking was required, on average, seven years after symptoms onset.

**Table 1:** Clinical characteristics of 20 LGMD2 patients clinically examined in the village.

LGMD2A	N = 20
**Male/Female**	10/10
**Muscle weakness**	20
**Affectation of the pelvic girdle**	20
**Scoliosis**	12
**Pseudohypertrophy of gastrocnemius**	12
**Winged scapulae**	12
**Lordosis**	10
**Affectation of shoulder girdle**	7

### Molecular analysis

**Fig 2:**
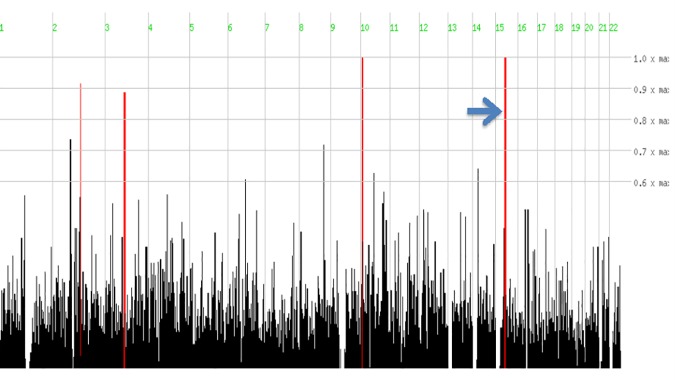
Genome-wide homozygosity analysis in LGMD2. Genotypes obtained with the Affymetrix 250K SNPs chip were analyzed with the HomozygosityMapper software for the identification of large regions of homozygosity. Top bars indicate homozygous regions identified in pooled DNA from three affected patients. As shown in the screen shot, two chromosomal regions of maximal homozygosity were identified. The largest region (6.6 Mb) corresponded to chromosome 15q (pointed with a blue arrow), from nucleotide 39,911,352 to nucleotide 46,517,283, and includes the *CAPN3* gene.

**Fig 3:**
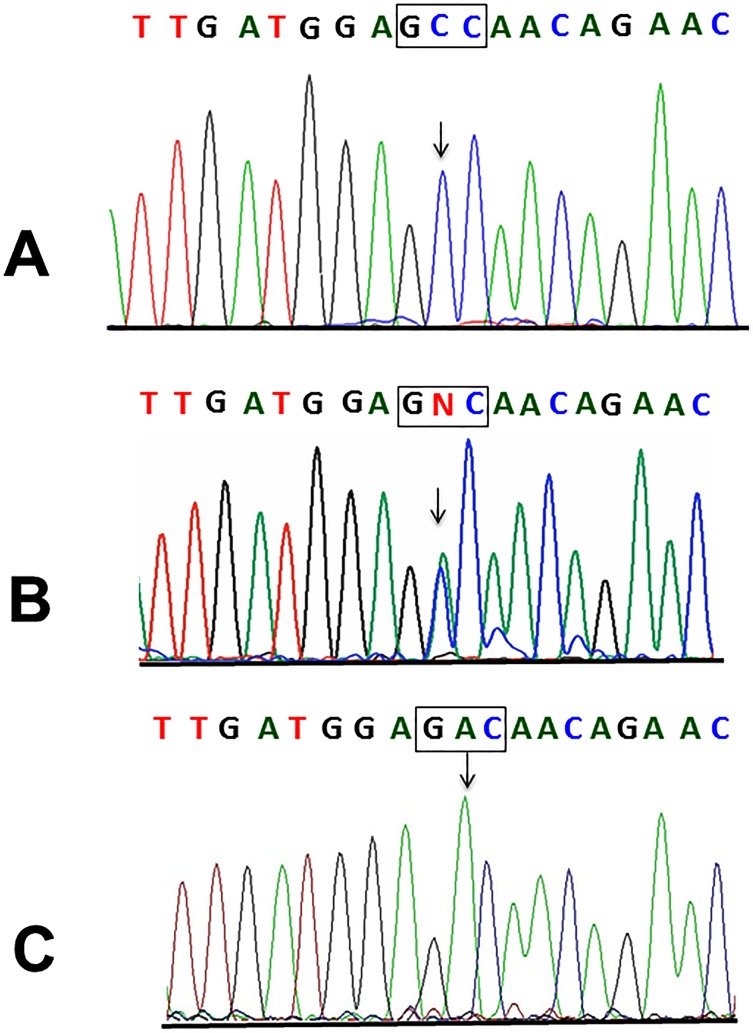
Partial nucleotide sequence of the CAPN3 gene. (A) Normal sequence showing homozygosity for the wild type GCC (alanine) codon at position 116. (B) Sequence from a heterozygous (GCC/GAC) subject (C) Sequence in DNA from a homozygous mutant (GAC/GAC) LGMD2 subject. The arrow indicates the mutated nucleotide (c.348C>A).

**Fig 4:**
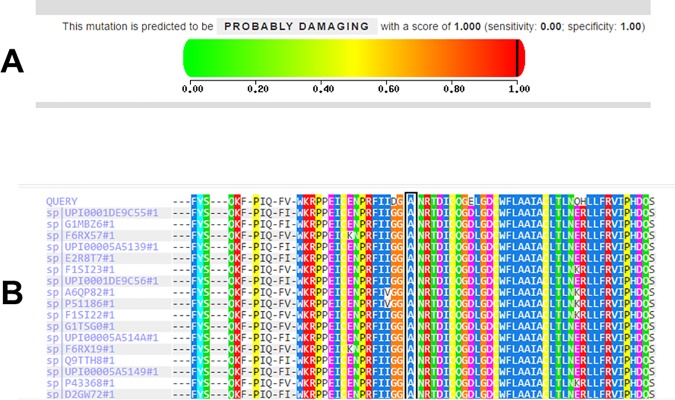
In silico analyses of the p.Ala116Asp mutation in CAPN3. (A) Pathogenicity prediction analysis by Polyphen-2 indicates that the missense substitution is a damaging mutation. (B) Phylogenetic comparison of CAPN3 proteins. Alanine 116 is strictly conserved in proteins from different species including Equus caballus (horse), Canis familiaris (dog), Sus scrofa (wild pig) Bos taurus (cow), Loxodonta africana (elephant), Oryctolagus cuniculus (rabbit), and Macaca mulatta (macaque), among many others.

Genome-wide SNP data analyzed by *HomozygosityMapper* revealed two extended (>2 Mb) regions of homozygosity (Fig 2). At chromosome 10p15, a 2.5 Mb region of homozygosity was identified (from nucleotide 4,618,822 to nucleotide 7,112,076). However, no genes related to muscular diseases were present in such segment (see S1 Table). The second homozygous region was located at chromosome 15q15, spanned 6.6 Mb from nucleotide 39,911,352 to nucleotide 46,517,283, and contained 11 known genes. The Calpain 3 (*CAPN3*) gene, responsible for autosomal recessive LGMD type 2A was located within this candidate interval. Direct nucleotide sequencing of *CAPN3* in DNA from 4 affected subjects revealed that all of them harbour a novel homozygous cytosine to adenine transversion at nucleotide 348 in exon 2. The c.348C>A mutation predicts a novel substitution from alanine (GCC) to aspartic acid (GAC) at amino acid 116 (p.Ala116Asp) of the CAPN3 protein (Fig 3). The p.Ala116Asp mutation was absent from exome variant databases as ExAC and Exome variant Server and was also absent from a set of 200 ethnically matched control alleles. The *in silico* prediction of mutation pathogenicity indicates that p.Ala116Asp is a protein damaging substitution by both Polyphen II and SIFT algorithms (Fig 4A). A comparative sequence alignment of CAPN3 proteins demonstrated a strict phylogenetic conservation of alanine 116 among numerous species (Fig 4B). Genotyping analysis disclosed that while the c.348C>A mutation was present in homozygous state in DNA from 19 out of 20 LGMD2 cases who were available for molecular study, it was heterozygous in a single affected subject. This finding raised the possibility of compound heterozygosity in this patient and therefore the entire *CAPN3* gene was sequenced in DNA from such case. As a result, a heterozygous deletion/insertion c.2362AG>TCATCT was identified in *CAPN3* exon 22. This is a previously described LGMD2A mutation that predicts a shift of the reading frame and the introduction of a premature stop codon that truncates the CAPN3 protein [9]. No apparent differences regarding age of symptoms onset or clinical severity were observed between CAPN3 homozygous patients and the single compound heterozygous case.

### Functional evaluation of CAPN3 protein

**Fig 5:**
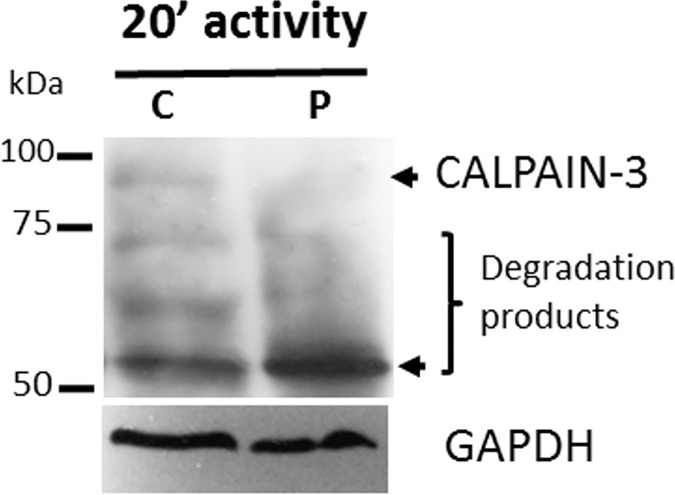
Inmunoblot analysis of CAPN3 in muscle biopsy from a LGMD2A patient with the novel p.Ala116Asp mutation. Western blot on skeletal muscle of normal control (C) and an affected patient (P) at 20 min post-incubation in saline solution indicates preserved autocatalytic activity of CAPN3 in both samples. However, both the absence of the 94 kDa band (top arrow) and the increased density of the degradation 60 kDa band (bottom arrow) suggest an increased autolytic activity in patient’s sample. Approximate molecular masses (kDa) are shown as a guide on the left. The range of protein degradation products is indicated. GAPDH was used as internal control.

Autocatalytic activity of calpain-3 protein was evaluated in muscle biopsy from a LGMD2A patient homozygous for the novel p.Ala116Asp CAPN3 mutation. Immunoblot analysis with Calp3c/12A2 antibody, that identifies bands of 94 kDa (full-length CAPN3) and 60 kDa (degradation product), showed that autocatalytic activity is increased in protein sample from the patient as indicated by both the absence of the 94 kDa band and the increase of the degradation 60 kDa band (Fig 5). As shown, the intensity of the degradation product (60 kDa band) is increased in the patient’s sample compared to control sample, suggesting higher catalytic activity. The same pattern was observed using CAPN3 antibodies from another supplier (Calp3d/2C4; Abcam, Cambridge, UK) (data not shown).

### *CAPN3* mutation carrier frequency

Estimation of mutation carrier frequency was conducted in a random sample of non-LGMD people in the community. Genomic DNAs from 401 adult individuals without muscular disease were analyzed for the presence of the *CAPN3* c.348C>A mutation. A total of 38 subjects (9.47%; CI: 6.89%-12.65%) demonstrated heterozygosity for the mutation, yielding a population carrier frequency of 1 in 11. The theoretical probability of a marriage between two heterozygotes living in this population (considering that the prevalence of carriers in reproductive age was 11.48%) was calculated to be 1 of every 75 marriages.

## Discussion

Mexican mestizos are a recently admixed population composed of Amerindian, European, and, to a lesser extent, African ancestry. Although this ethnic admixture generated considerable genetic heterogeneity between subpopulations from different regions throughout the country [23], the practice of consanguineous marriages, which is still common in many communities, reduces genetic variation and increases the chances of recessive disease. In this work, the clinical, epidemiological, and molecular characterization of an isolated Mexican population with a high incidence of LGMD type 2A was performed. LGMD2 is a clinically and genetically heterogeneous group of myopathies with substantial prevalence variation in different geographical regions. Previously, the ethnic origin of patients was suggested as a feature to be taken into account for helping in the clinical distinction between LGMD subtypes (Reviewed in [11]). To date, very few investigations on the clinical and molecular features of Mexican LGMD patients have been performed. In a recent report from Mexico, Gomez-Diaz et al. used immunofluorescence staining to analyze muscle biopsies from 212 patients with various muscular dystrophies and identified that 18% of them corresponded to dysferlinopathies while 11% were calpainopathies [24].

Several population isolates with a high frequency of LGMD2 have been described (reviewed in [11]). Most of these aggregates arise from founder mutation effects favored by limited genetic interchange in endogamic groups. In such populations, genomic techniques exploiting the genetic uniformity of affected individuals could facilitate molecular diagnosis of genetically heterogeneous diseases as autosomal recessive LGMD [18]. Particularly, homozygosity mapping, also known as autozygosity mapping, is a method that takes advantage of the fact that inbred affected individuals are likely to have inherited two recessive copies of the disease allele from a common ancestor, i.e. two identical‐by‐descent (IBD) alleles [25]. Homozygosity mapping has been shown to be an effective method to identify disease-causing mutations in LGMD2 patients from consanguineous families [26–28]. In the present work the involved LGMD2 locus was efficiently identified using genome wide homozygosity mapping. After localization of the locus and identification of *CAPN3* as a candidate gene, a novel p.Ala116Asp causative mutation was recognized in this gene. This mutation affects a phylogenetically conserved alanine residue located at the active cysteine protease domain of CAPN3 protein.

Western blot analysis of CAPN3 in muscle from a patient with this novel mutation suggested that the mutant protein does not lose its autolytic activity but rather it is probably increased. However, it should be noted that as a LGMD2A diagnostic tool, CAPN3 western blotting have a high rate of false positives and negatives, with an estimated sensitivity of 52.5% and a specificity of 87.8% [29].

As anticipated, considering the isolation of the population, the mutation was demonstrated to be identical and homozygous in all affected patients (founder effect). Unexpectedly, however, one clinically affected adult patient demonstrated heterozygosity for the p.Ala116Asp mutation. This finding prompted us to screen the entire coding sequence of *CAPN3* allowing the identification of a second heterozygous mutation, c.2362AG>TCATCT, at exon 22. This mutation has been previously reported [9] and is the most common LGMD2A causing mutation in patients from the Basque country in Spain [29, 30].

Interestingly, the age of the c.2362AG>TCATCT mutation has been estimated to be 50 generations (i.e., 1,250 years), which is before the arrival of the Spaniards in Mexico [31]. Thus, given the considerable genetic admixture between Spaniards and native Mexicans starting at century XVI, it is probable that this CAPN3 mutation was brought from Europe. Nonetheless, no history of immigrant ancestors could be recorded in the family from our compound heterozygous LGMD2A patient.

Although unanticipated in our study, the identification of more than one pathogenic allele in population isolates segregating LGMD2A has also been demonstrated in previous reports. Particularly, in an isolated population at the La Réunion Island in the Indian Ocean, several LGMD2A-causing alleles were identified by Richard et al. [9]. This was an unexpected result given the fact that affected patients of La Réunion all belong to a small genetic isolate, presumed to derive from a single ancestor who immigrated to this island in the 1670s. These patients were all thus expected to carry the same LGMD2A mutation. The presence of at least six different LGMD2A mutations among patients from the La Réunion island is known as the “Reunion paradox” [9, 32, 33]. Interestingly, the c.2362AG>TCATCT mutation identified by us in one out of 40 *CAPN3* alleles from LGMD2 has been also recognized in families from La Reunion Island and from Brazil [9]. The most plausible explanation for the occurrence of multiple disease alleles in “closed” populations is the occurrence of sporadic waves of immigration with introduction of additional founders [34].

Another important aspect that arose from our investigation is the high carrier frequency of the founder p.Ala116Asp CAPN3 mutation in village inhabitants, which was estimated to be of about 1 in 11. This figure sharply contrasts with previous data on carrier frequency for other *CAPN3* mutations which range from 1 in 103 to 1 in 160 in other populations [10, 34]. It is worthy to note that in a previous study from our group in this Mexican village we demonstrated a high incidence of a severe autosomal recessive eye malformation known as sclerocornea [35]. A homozygous founder mutation in the *FOXE3* gene was proved to underlie the disease and a mutation carrier frequency of 1 in 40 was estimated. These data indicates that LGMD2A carriers are almost 4 times more frequent that sclerocornea carriers in this highly inbred population. In such preceding study we were able to estimate that the “age” of entry of the sclerocornea-causing mutation in the village was approximately 106 to 138 years [33]. Thus, as the *CAPN3* carrier frequency is higher than the *FOXE3* carrier frequency, it is probable that the LGMD2A-causing mutation arrived before to this population isolate.

In conclusion, we describe a cluster of patients with LGMD type 2A arising from a novel CAPN3 p.Ala166Asp founder mutation in a small village from central Mexico. Evidence of allelic heterogeneity was provided by the recognition of a second *CAPN3* pathogenic allele in a single affected individual from this closed community. Our study provides an additional example of genetic isolation causing a high prevalence of LGMD2 and the successful molecular characterization of the disease by means of genomic analysis methods as homozygosity mapping. The identification of a very high carrier frequency of the LGMD2 causing mutation has implications for more rational genetic counseling in this community.

### *In silico* resources

Polyphen-II, http://genetics.bwh.harvard.edu/pph/

SIFT, http://sift.jcvi.org/

HomozygosityMapper, www.homozygositymapper.org

GeneDistiller software, www.genedistiller.org

Exac, http://exac.broadinstitute.org/

Human Gene Mutation Database, HGMD http://www.hgmd.cf.ac.uk/ac/index.php

Exome variant Server, http://evs.gs.washington.edu/EVS/

## Supporting Information

S1 Fig Data on population structure. (TIF)

S1 Table List of genes located within the 2.5 Mb region of homozygosity at chromosome 10p. (DOCX)
