# Supplementary figures and images for: Epidemiological and Molecular Characterization of a Mexican Population Isolate with High Prevalence of Limb-Girdle Muscular Dystrophy Type 2A Due to a Novel Calpain-3 Mutation
Source: PLoS One. 2017 Jan 19;12(1):e0170280. doi: 10.1371/journal.pone.0170280 (PMC5245889; doi:10.1371/journal.pone.0170280)

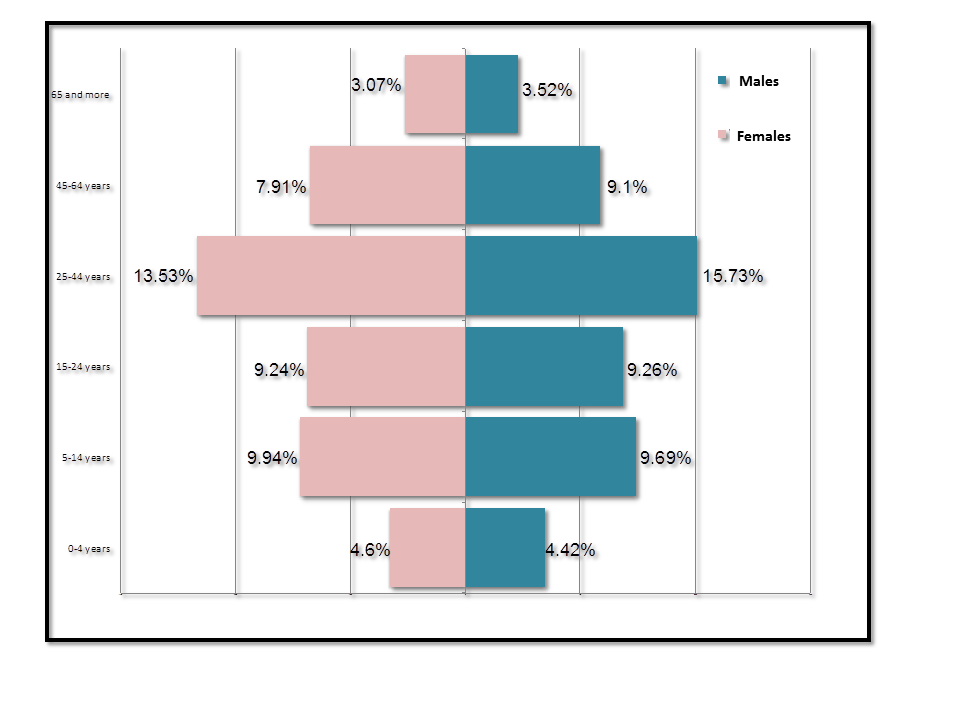

Supplement: S1 Fig — (TIF) [file pone.0170280.s001.tif]
