# Supplementary material for: Epidemiological and Molecular Characterization of a Mexican Population Isolate with High Prevalence of Limb-Girdle Muscular Dystrophy Type 2A Due to a Novel Calpain-3 Mutation
Source: PLoS One. 2017 Jan 19;12(1):e0170280. doi: 10.1371/journal.pone.0170280 (PMC5245889; doi:10.1371/journal.pone.0170280)
Supplement: S1 Table — (DOCX) [file pone.0170280.s002.docx]

**Supplementary table.- List of genes located within the 2.5 Mb region of homozygosity at chromosome 10p**

| **Gene** | **Associated phenotype in humans** |
| --- | --- |
| AKR1E2 | None |
| AKR1C1 | None |
| AKR1C2 | 46,XY sex reversal |
| AKR1C3 | Polycystic ovary syndrome |
| AKR1C4 | 46,XY sex reversal (modifier) |
| UCN3 | None |
| TUBAL3 | None |
| NET1 | None |
| CALML5 | None |
| CALML3 | None |
| ASB13 | None |
| FAM208B | None |
| GDI2 | None |
| ANKRD16 | None |
| FBXO18 | None |
| IL15RA | None |
| IL2RA | Immunodeficiency with lymphoproliferation and autoimmunity |
| RBM17 | None |
| PFKFB3 | None |
| PRKCQ | None |
